# Functional role of eukaryotic translation initiation factor 4 gamma 1 (EIF4G1) in NSCLC

**DOI:** 10.18632/oncotarget.8168

**Published:** 2016-03-18

**Authors:** Yueyu Cao, Mengdan Wei, Bing Li, Yali Liu, Ying Lu, Zhipeng Tang, Tianbao Lu, Yujiao Yin, Zhiqiang Qin, Zengguang Xu

**Affiliations:** ^1^ Department of Oncology, Shanghai East Hospital, Dalian Medical University, Shanghai 200120, China; ^2^ Department of Oncology, Shanghai East Hospital, Tongji University School of Medicine, Shanghai 200120, China; ^3^ Research Center for Translational Medicine and Key Laboratory of Arrhythmias, Shanghai East Hospital, Tongji University School of Medicine, Shanghai 200120, China; ^4^ Departments of Microbiology/Immunology/Parasitology, Louisiana State University Health Sciences Center, Louisiana Cancer Research Center, New Orleans, LA 70112, USA

**Keywords:** EIF4G1, USP10, NSCLC, lung cancer

## Abstract

Eukaryotic translation initiation factor 4 gamma 1(EIF4G1) is related to tumorigenesis and tumor progression. However, its role and the underlying mechanisms in the regulation of tumor development in non–small cell lung cancers (NSCLC) remain largely unknown. Here we report that the levels of EIF4G1 expression are much higher in NSCLC cell lines and tumor tissues than those in the normal lung cells and adjacent normal tissues from the same patients. Using shRNA to knock down EIF4G1 expression stably, we found EIF4G1 required for NSCLC cell proliferation, anchorage-independent growth, migration and invasion. Furthermore, silencing of EIF4G1 induces NSCLC cell apoptosis and causes G0/G1 cell cycle arrest. To identify the partner protein network of EIF4G1 in NSCLC cells, we found that Ubiquitin-specific protease 10 (USP10) can directly interacts with EIF4G1, while acting as a negative regulator for EIF4G1-mediated functions. Together, our results indicate that EIF4G1 functions as an oncoprotein during NSCLC development, which may represent a novel and promising therapeutic target in lung cancer.

## INTRODUCTION

Lung cancer is a common malignant disease and the leading cause of cancer-related death in the world [1], which falls into the broad categories of non–small cell lung cancers (NSCLC, encompassing lung adenocarcinomas, squamous cell lung carcinomas and large cell carcinomas [2]) and SCLC [3]. Notably, NSCLC accounts for approximately 80%~85% of lung cancers [4]. Although the conventional treatment (surgery and chemotherapy) of NSCLC advances, NSCLC has a dismal 5-year survival rate of 16% due to the majority of patients present at the diagnosis with advanced stages [5, 6], as well as high incidence of recurrence [7]. In recent years, better understanding of the molecular biology of NSCLC has led to a revolution in the treatment of these neoplasms [8]. Searching for specific mutations in individual cases so as to provide the most effective treatment with the least possible occurrence of side effects is a reality [9]. The identification of oncogenic activation of particular tyrosine kinases (TKs) in some patients with advanced NSCLC in particular those mutations in epidermal growth factor receptor (EGFR) [10-12] have demonstrated that detection of such mutations in the plasma of newly diagnosed NSCLC patients is feasible [13], and led to the development of personalized medicine for these patients. Even though initial therapy with an EGFR TK inhibitor is quite effective, acquired resistance still develops after 10–15 months treatment [14]. So there is an urgent need for further understanding the molecular mechanisms of lung cancer tumorigenesis and identifying new therapeutic targets to improve the prognosis of cancer patients [15].

Recent studies have reported that the eukaryotic translation initiation factor 4 gamma 1 (EIF4G1) is related to tumorigenesis and tumor progression in breast cancer [16]. EIF4G1 participates in protein translation by serving as a scaffold and interacting with several other initiation factors [17]. Human EIF4G1 was first isolated as part of a large protein complex that could restore protein synthesis [18] and the complex was later identified as EIF4F in which the initiation factor EIF4G1 associates with EIF4E and EIF4A [19]. The function of the complex EIF4F is to recruits ribosomes to the capped end of mRNA to initiate cap-dependent translation [20]. Over the last two decades, the translation initiation complex EIF4F has been shown to play important roles in oncogenesis [21-23]. In addition, recent studies have found that EIF4G1 is overexpressed in a variety of cancers including nasopharyngeal cancer [24], squamous cell carcinoma [25] and breast cancer [26]. Although published literature have demonstrated that EIF4G1 plays important roles in cancer pathogenesis, progression, and treatment [27], its functional role in NSCLC remains largely unknown. One recent study reported that inhibition of c-jun N-terminal kinase (JNK) leads to a decrease in formation of the cap-dependent translation complex, EIF4F, in NSCLC cells [28].

In the current study, we found that EIF4G1 is required for NSCLC cell survival and proliferation, and it also acts as an oncoprotein to promote lung cancer cell malignant behaviors. More interestingly, we found that the Ubiquitin-specific protease 10 (USP10) can interact with EIF4G1 and functions as a negative regulator of EIF4G1 to control NSCLC cell survival.

## RESULTS

### EIF4G1 expression in NSCLC cell lines and tumor specimens

**Figure 1:**
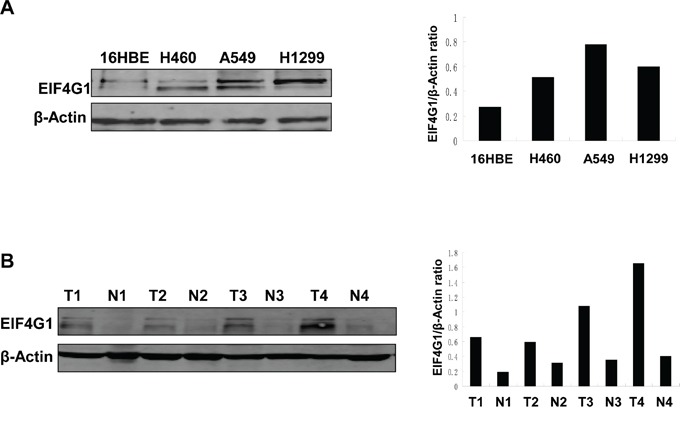
The increased levels of EIF4G1 expression in NSCLC cell lines and tumor tissues **A.** EIF4G1 expression in 3 NSCLC cell lines (H460, A549, H1299) and normal lung epithelial cell line (16HBE) was detected and compared by immunoblots. **B.** Representative immunoblot results for EIF4G1 expression in tumor tissues (T) and adjacent normal tissues (N) collected from 4 NSCLC patients were shown. The protein band density was quantitated using Image-J software.

We first detected the expression of EIF4G1 in several NSCLC cell lines and collected tumor tissues. We found that EIF4G1 expression was greatly increased in 3 NSCLC cell lines (H460, A549, H1299) when compared to normal human bronchial epithelial cell line, 16HBE (Figure 1A). Furthermore, we found that EIF4G1 expression was significantly up-regulated in most NSCLC tumor tissues we detected (13/18, 72%) when compared to the respective adjacent normal lung tissue from the same patients. Representative immunoblot results were shown in Figure 1B. These data suggests that EIF4G1 may have important role in NSCLC development.

### EIF4G1 is required for NSCLC cell proliferation and anchorage-independent growth

**Figure 2:**
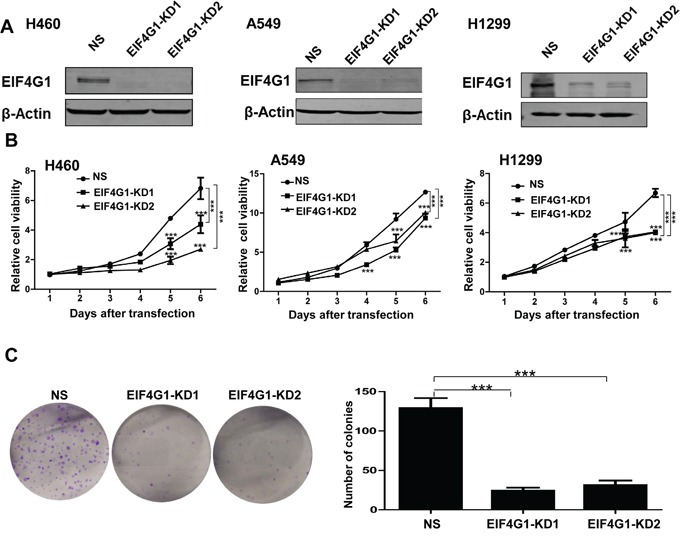
EIF4G1 is required for NSCLC cell proliferation and anchorage-independent growth **A.** The expression of EIF4G1 in stably “knock-down” NSCLC cells by using lentiviral vector containing 2 shRNA specifically targeting EIF4G1 (EIF4G1-KD1 and KD2) was detected by immunoblots. A non-silencing (NS)-shRNA was used as a negative control. **B.** The cell growth of stably EIF4G1 “knock-down” NSCLC cells and control (NS) was measured and compared by using the CCK8 assays. **C.** The anchorage-independent growth abilities of stably EIF4G1 “knock-down” A549 cells and control (NS) were measured by using colony formation assays. Error bars represent the S.E.M. for 3 independent experiments. ***=*p*<0.001.

To study the functional role of EIF4G1 in NSCLC, we first directly silenced it by using lentiviral vector containing 2 shRNA specifically targeting EIF4G1 (EIF4G1-KD1 and KD2) to obtain stably “knock-down” H460, A549, H1299 cells. A non-silencing (NS)-shRNA was used as a negative control. Silencing of EIF4G1 dramatically reduced its expression when compared to the control cells in these tumor cells (Figure 2A). Using Cell Counting Kit-8 (CCK8) assay, we found that silencing of EIF4G1 significantly reduced all the 3 NSCLC cell lines growth/proliferation (Figure 2B). Next, we tested the effects of “knock-down” EIF4G1 on anchorage-independent growth abilities of NSCLC cells by using the colony formation assay. Our data indicated that silencing of EIF4G1 effectively reduced the colony formation of A549 and H1299 cells on soft agar (Figure 2C and S1).

### Targeting EIF4G1 induces NSCLC cell apoptosis and cell cycle arrest

**Figure 3:**
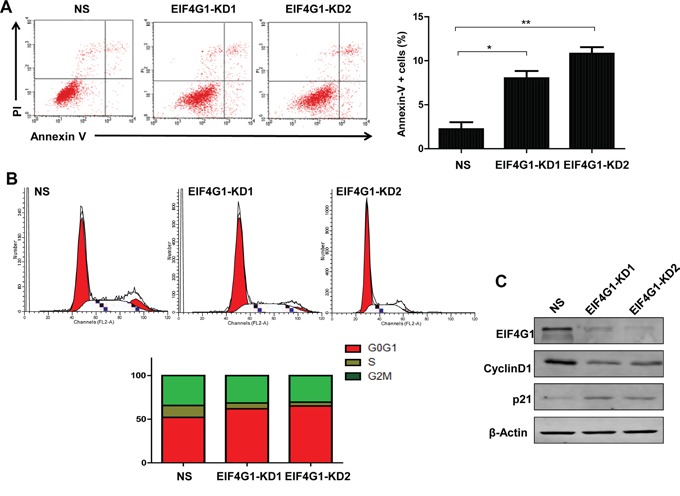
Targeting EIF4G1 induces NSCLC cell apoptosis and G0/G1 cell cycle arrest **A.** Cell apoptosis of stably EIF4G1 “knock-down” A549 cells (EIF4G1-KD1 and KD2) and control (NS) was determined by Annexin-V/PI staining and flow cytometry analysis. Error bars represent the S.E.M. for 3 independent experiments. *=*p*<0.05, **=*p*<0.01. **B-C.** Cell cycle was determined by PI staining and flow cytometry analysis. Protein expression was detected by immunoblots.

To further investigate the mechanisms of EIF4G1 regulating NSCLC cell growth, we found that silencing of EIF4G1 induced A549 cell apoptosis by using Annexin-V/PI staining and flow cytometry analysis (Figure 3A). We got similar results in H460 and H1299 cell lines (data not shown). Furthermore, we found that silencing of EIF4G1caused G0/G1 cell cycle arrest in A549 and H1299 cell lines (Figure 3B and S2). We further confirmed that silencing of EIF4G1 affected several cell cycle/check point regulatory proteins, including increased the expression of p21 while reduced CyclinD1 expression in A549 (Figure 3C) and H1299 cells (data not shown). Taken together, our data demonstrate that cell apoptosis and cell cycle processes are involved in EIF4G1 regulation of NSCLC growth.

### Targeting EIF4G1 blocks NSCLC cell migration and invasion

**Figure 4:**
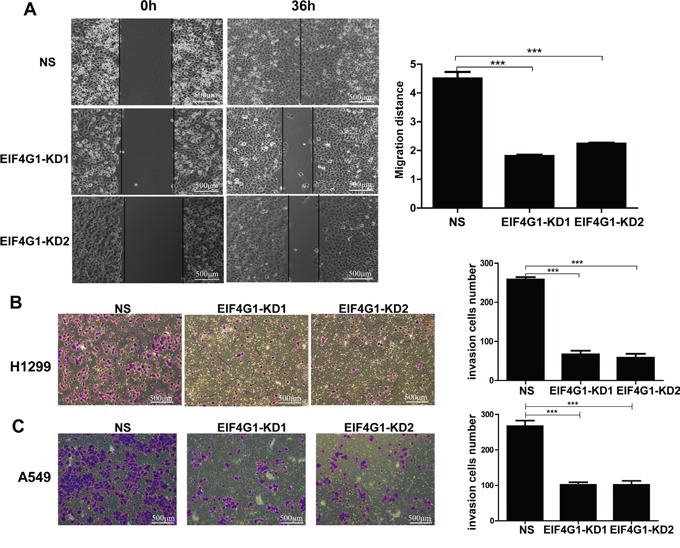
Targeting EIF4G1 blocks NSCLC cell migration and invasion **A.** Cell migration of stably EIF4G1 “knock-down” H1299 cells (EIF4G1-KD1 and KD2) and control (NS) were examined using wound healing assays. **B-C.** Cell invasion of stably EIF4G1 “knock-down” H1299 and A549 cells and control (NS) were measured using transwell assays. Bars, 500μm. Error bars represent the S.E.M. for 3 independent experiments. ***=*p*<0.001.

We also tested the functional role of EIF4G1 in other malignant behaviors of NSCLC cells such as migration and invasion, which are important to lung cancer development and metastasis. By using wound healing assay, we found that silencing of EIF4G1 effectively blocked H1299 cell migration (Figure 4A), as well as A549 cells (data not shown). By using transwell assay, we found that silencing of EIF4G1 significantly reduced cell invasiveness of both H1299 and A549 cells (Figure 4B-4C). These data indicate that EIF4G1 may act as an oncoprotein contributed to NSCLC development.

### USP10 is a potential partner protein involved in EIF4G1-mediated functions in NSCLC

**Figure 5:**
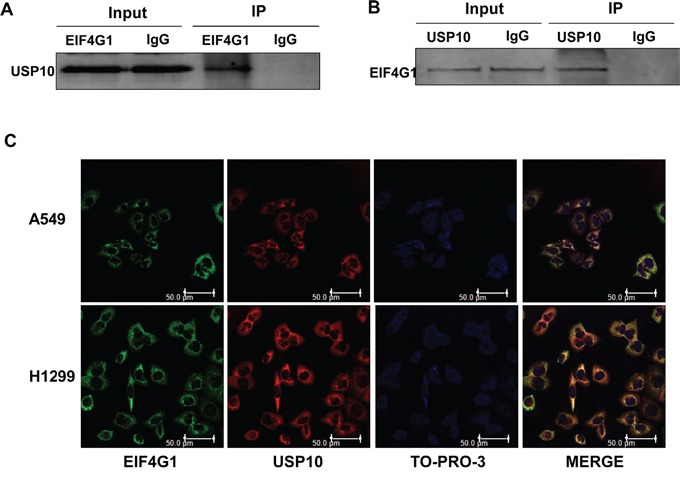
The interaction of EIF4G1 and USP10 in NSCLC **A-B.** Immunoprecipitation assays were performed using anti-EIF4G1 and anti-USP10 antibodies (IgG is a negative control) as described in the Methods. **C.** The co-localization of EIF4G1 and USP10 in A549 or H1299 cells was confirmed using immunofluorescence assays. Bars, 50μm.

To identify the interaction proteins with EIF4G1 by using tandem affinity purification combined with mass spectrometry (TAP-MS) screening approach, we found that at least one protein, Ubiquitin-specific protease 10 (USP10), can directly interact with EIF4G1 in NSCLC cell lines. USPs belong to a complex family of deubiquitinating enzymes that specifically cleave ubiquitin conjugates on a great variety of substrates, thereby, USPs regulate the production and recycling of ubiquitin and are critically involved in the control of cell growth, differentiation, and apoptosis [29, 30]. USP10 consists of 798 amino acids and belongs to the USPs family of cysteine proteases [31]. It has been demonstrated that USP10 is involved in modulating the androgen receptor (AR) function [32], and regulating vesicular transport and trafficking of membrane proteins [33]. By using co-immunoprecipitation and immunofluorescence approaches, we further confirmed the interaction of EIF4G1 and USP10 in H1299 cells (Figure 5). We also observed similar results in A549 cells (data not shown).

**Figure 6:**
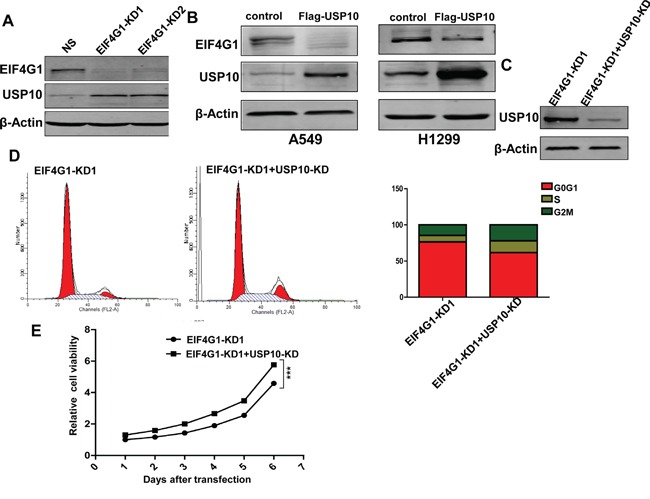
USP10 is involved in EIF4G1-mediated functions as a potential negative regulator **A.** The expression of USP10 in stably EIF4G1 “knock-down” A549 cells (EIF4G1-KD1 and KD2) and control (NS) was determined by immunoblots. **B.** A549 and H1299 cells were transfected with for the recombinant USP10 vector (Flag-USP10) or control vector for 48 h, then protein expression was measured by immunoblots. **C-E.** A549 cells were stably transfected with shRNA-EIF4G1 (EIF4G1-KD1) together with or without shRNA-USP10 (USP10-KD), then protein expression, cell cycle and growth were analyzed as described previously. Error bars represent the S.E.M. for 3 independent experiments. ***=*p*<0.001.

Interestingly, we found that silencing of EIF4G1 resulted in the up-regulation of USP10 in A549 cells (Figure 6A). To confirm this result, we overexpressed USP10 in A549 and H1299, and which inversely down-regulating EIF4G1 expression in both cell lines (Figure 6B). We also detected and compared the expressional level of USP10 in NSCLC tumor tissue and the respective adjacent normal lung tissue from 18 cancer patients. However, we found that only 50% of patients (9/18) have the reduced expression of USP10 in tumor tissues, implying that additional factors are involved in regulation of USP10 expression in NSCLC. Representative immunoblot results were shown in Figure S3. We next knocked down USP10 by RNAi and found it partially rescued A549 cells from G0/G1 cell cycle arrest and growth inhibition caused by silencing of EIF4G1 (Figure 6C-6E). Taken together, our data indicate that USP10 may act as a partner protein but negative regulator involved in EIF4G1-mediated functions within NSCLC cells.

## DISCUSSION

Lung cancer in particular NSCLC has the highest mortality in both sexes worldwide [4]. Recent studies have provided the proof-of-principle that NSCLC is represented by a group of molecularly heterogeneous diseases [34]. Several genetic mutations, such as EGFR, HER2, MET and fusion oncogenes involving anaplastic lymphoma kinase (ALK), ROS1 and RET, have been identified and validated as oncogenic drivers, able to determine the development and maintenance of specific subclasses of NSCLC [35, 36]. Patients with tumor harboring driver mutations are characterized by improved prognosis, response and outcome to targeted therapy or personalized therapy [37]. However, most patients have advanced NSCLC at diagnosis and despite recent advancements over the last 20 years, their 5-year survival rate is only 5% [37]. Therefore, it is still an urgent need to identify new drug target and develop more effective therapeutic strategies for NSCLC. EIF4G1 has been found overexpressed in a variety of cancers, and playing important roles in cancer pathogenesis, progression, and treatment [24-27]. To our knowledge, its role in NSCLC has not been extensively investigated. Here we first time report that EIF4G1 expression is greatly up-regulated in NSCLC cell lines and tumor tissues when compared to those normal controls. Functional assays indicate that EIF4G1 is involved in NSCLC cell survival/proliferation, and multiple malignant behaviors for tumor development.

Interestingly, several EIF4G1-targeted molecules have been shown promising in experimental cancer treatment. For example, 4EGI-1, an inhibitor of the interaction between translation initiation factors EIF4E1 and EIF4G1 effectively inhibits breast cancer stem cells (CSCs) through selectively reducing translation persistent in breast CSCs [38, 39]. Furthermore, 4EGI-1 isomers suppress breast CSC tumor angiogenesis and tumor growth *in vivo* [38]. Another small molecule SBI-0640756 (SBI-756), a first-in-class inhibitor that targets EIF4G1 and disrupts the EIF4F complex, can effectively inhibit the growth of NRAS, BRAF, and NF1-mutant melanomas *in vitro* and delay the onset and reduce the incidence of Nras/Ink4a melanomas *in vivo* [40]. Future studies will focus on whether these molecules can also be used for NSCLC treatment.

By using TAP-MS screening approach, we first time demonstrate that USP10 is a partner protein directly interacting with EIF4G1, and acts as a negative regulator for EIF4G1-mediated functions in NSCLC. However, the underlying mechanisms for USP10 interacting with EIF4G1 and regulatory functions in NSCLC still require further investigation. We are now constructing varied fragment mutants for USP10 and EIF4G1, in order to determine the key domain or amino acid residues required for protein-protein interaction. In fact, USP10 has abnormal expression and plays important roles in a variety of tumor cells growth such as breast cancer [41], glioblastoma multiforme (GBM) [42], adult T-cell leukemia (ATL) [43] and pancreatic cancer [44], although the underlying mechanisms remain largely unknown. Interestingly, one recent study reports that microRNA-191 can promote pancreatic cancer progression by targeting USP10 [45]. In addition, USP10 has been linked to several intracellular signaling pathways for its cellular functions. For example, USP10 can inhibit genotoxic NF-κB activation through monocyte chemotactic protein-1-induced protein-1 (MCPIP1)-facilitated deubiquitination of NEMO [46]. USP10 has been found to directly deubiquitinate p53 and to be an essential regulator of the p53 stability, and it can act as either a tumor suppressor or an oncoprotein, depending on wild type (wt) p53 or mutant p53 background [47]. Recent study have found the downregulation of USP10 in a high percentage of renal cell carcinoma (RCC) samples containing the wt p53, while the overexpressed USP10 in RCC cells with mutant p53 [47]. However, the role of p53 (wt or mutant) in the EIF4G1/USP10 interaction, expression and mediated functions in NSCLC requires further investigation. Taken together, our data indicate that EIF4G1 together with its partner proteins such as USP10 may represent a novel strategy for NSCLC treatment.

## MATERIALS AND METHODS

### Cell lines and cell culture

A total of 3 NSCLC cell lines (A549, H460, H1299) and normal human bronchial epithelial cell line (16HBE) were obtained from Shanghai Institutes for Biological Sciences (SIBS). NSCLC cell lines were cultured in RPMI-1640 medium (Coring) and 16HBE was maintained in Dulbecco's modified Eagle's medium (GIBCO). Both growth media were supplemented with 10% fetal bovine serum (GIBCO) and 1% penicillin & streptomycin (GIBCO).

### Carcinoma tissue samples

NSCLC samples and adjacent normal tissues were collected from 18 patients at Shanghai East hospital of Tongji University, Shanghai, China. Informed consent was obtained from each patient and the whole study was approved by the Committee on Human Rights in Research at Shanghai East hospital.

### Immunoblotting

Total cell lysates (20μg) were resolved by 10% SDS–PAGE, transferred to nitrocellulose membranes, and immunoblotted with antibodies for EIF4G1 (Cell Signaling), p21, CyclinD1, USP10 (Abcam) and β-Actin (Sigma) for loading controls. Immunoreactive bands were identified using an enhanced chemiluminescence reaction (Perkin-Elmer), and visualized by autoradiography.

### Immunofluorescence

Cells were seeded onto coverslips in a 6-well plate and fixed with 4% paraformaldehyde (w/v) for 30 min, and then were washed for 10 min with PBS and permeabilized with 0.2% (w/v) Triton X-100 in PBS for 5 min. Cells were blocked for 30 min in PBS containing 1% bovine serum albumin (BSA), then incubated overnight with the diluted primary EIF4G1and USP10 antibodies. After washed with PBS, cells were incubated for 1 h with secondary fluorescein isothiocyanate or tetra methyl rhodamine isothiocyanate-conjugated antibodies (Invitrogen). After additional washing, cells were stained with TO-PRO-3 (Thermo Fisher Scientific) and prepared for visualization using a Leica TCPS SP5 AOBS confocal microscope.

### CCK8 cell proliferation assay

The stable expressed shRNA-EIF4G1 or control cells (1,000 cells/well) were seeded in 96-well plates for 6 days, then 10 μL of Cell Counting Kit-8 reagent (CCK8, Sigma) was added, and the viability of the cells was measured at 450 nm using a microplate reader according to the manufacturer's instructions.

### Colony formation assay

The stable expressed shRNA-EIF4G1 or control cells (1,000 cells/well) were seeded in 6-well plates After 14 days, the cells were washed with PBS and fixed with fixation solution (methanol: glacial: acetic 1:1:8) for 10 min, then stained with crystal violet solution, and visible colonies were counted.

### Co-immunoprecipitation

Proteins extracted from H1299 cells were incubated respectively with a specific rabbit polyclonal antibody to EIF4G1 or a nonimmune rabbit IgG as the control at a final concentration of 1 g/mL overnight at 4°C. The immune complexes were pulled down by protein A/G agarose beads (Santa Cruz) and washed with PBS containing proteinase inhibitor for 3 times. The immunoprecipitated proteins were separated by SDS-PAGE and analyzed by immunoblotting using a USP10 antibody.

### Wound healing assay

The stable expressed shRNA-EIF4G1 or control cells were grown to full confluency in 12-well plates and incubated overnight in starvation medium. Cell monolayer was scratched with a sterile fine pipette tip and washed with medium to remove detached cells. Cells were kept in the incubator for 36 h, then the wound gap was observed under the microscope and cells were photographed.

### Flow cytometry

For apoptosis assays, the FITC-Annexin V and propidium iodide (PI) Apoptosis Detection Kit I (BD Pharmingen) was used. For cell cycle analysis, PEL cell pellets were fixed in 70% ethanol, and incubated at 4°C overnight. Cell pellets were re-suspended in 0.5 mL of 0.05 mg/mL Propidium Iodide (PI) plus 0.2 mg/mL RNaseA and incubated at 37°C for 30 min. Cell cycle distribution was analyzed on a FACS Calibur 4-color flow cytometer (BD Bioscience).

### Transwell invasion assay

The invasion assay was performed using 24-well Transwell chambers (8 μm; Corning). Following transfection, tumor cells were resuspended in serum-free RPMI 1640 medium and 5×10^4^ cells were seeded into the upper chambers covered with Matrigel (BD Bioscience), and 0.5 mL RPMI 1640 containing 20% FBS was added to the bottom chambers. After incubation for 48 h, the non-filtered cells were gently removed with a cotton swab. Filtered cells located on the lower side of the chamber were stained with 0.1% crystal violet (Sigma) and counted under the microscope.

### RNA interference

To establish stable EIF4G1 or USP10 knockdown cells, we used Dharmacon EIF4G1-shRNA1 (KD1), EIF4G1-shRNA2 (KD2), USP10-shRNA and a non-silencing (NS)-shRNA as a control.

### Statistical analysis

Significance for differences between experimental and control groups was determined using the two-tailed Student's t-test (SPSS v16.0), and p values <0.05 were considered significant.

## SUPPLEMENTARY FIGURES AND TABLES


