# Supplementary material for: Functional role of eukaryotic translation initiation factor 4 gamma 1 (EIF4G1) in NSCLC
Source: Oncotarget. 2016 Mar 18;7(17):24242–51. doi: 10.18632/oncotarget.8168 (PMC5029698; doi:10.18632/oncotarget.8168)
Supplement: Supplementary file 1 [file oncotarget-07-24242-s001.pdf]

SUPPLEMENTARY FIGURES

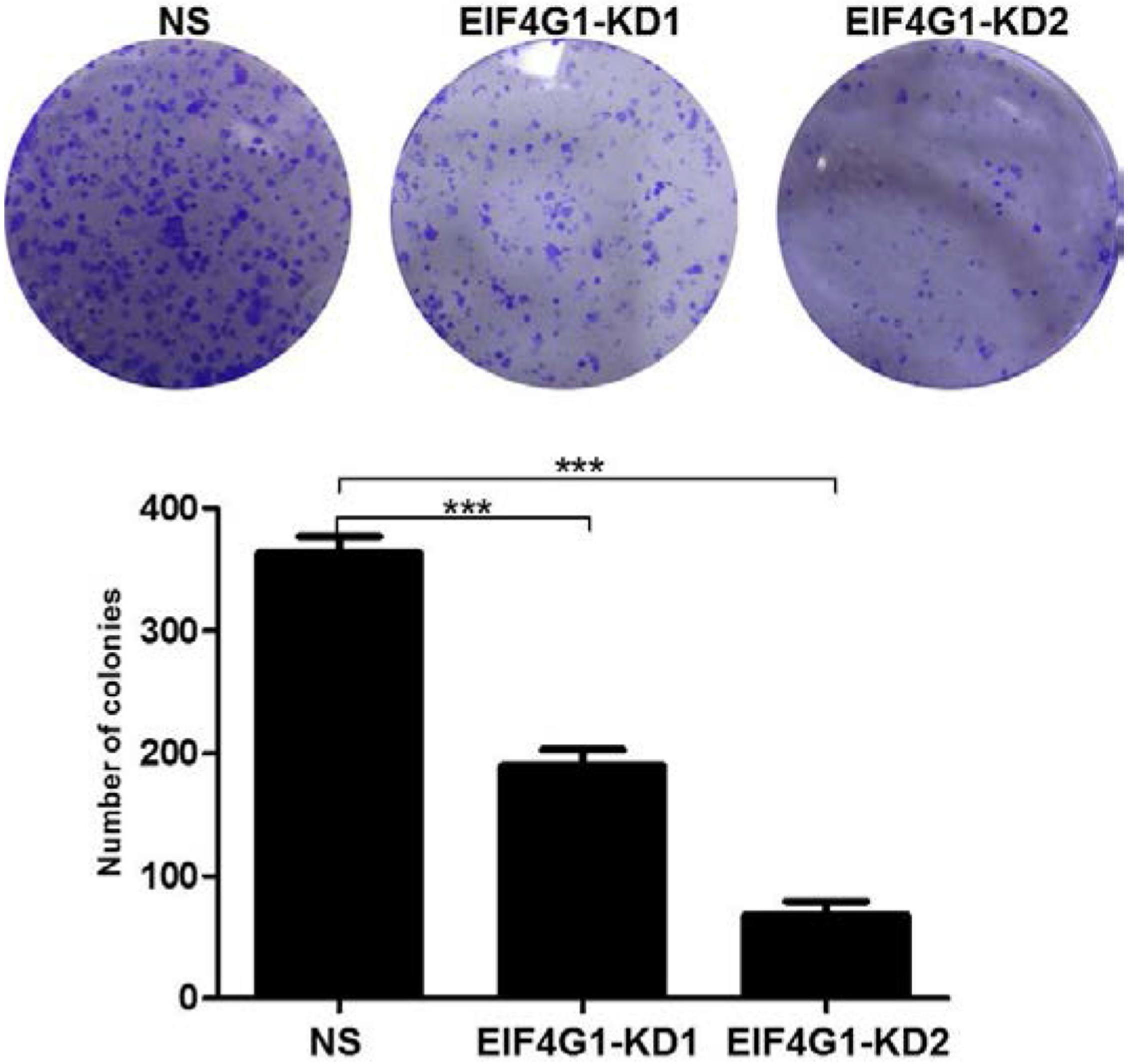

Supplementary Figure S1: EIF4G1 is required for H1299 anchorage-independent growth. The anchorage-independent growth abilities of stably EIF4G1 “knock-down” H1299 cells (EIF4G1-KD1 and KD2) and control (NS) were determined by colony formation assays. Error bars represent the S.E.M. for 3 independent experiments. ***=p<0.001.

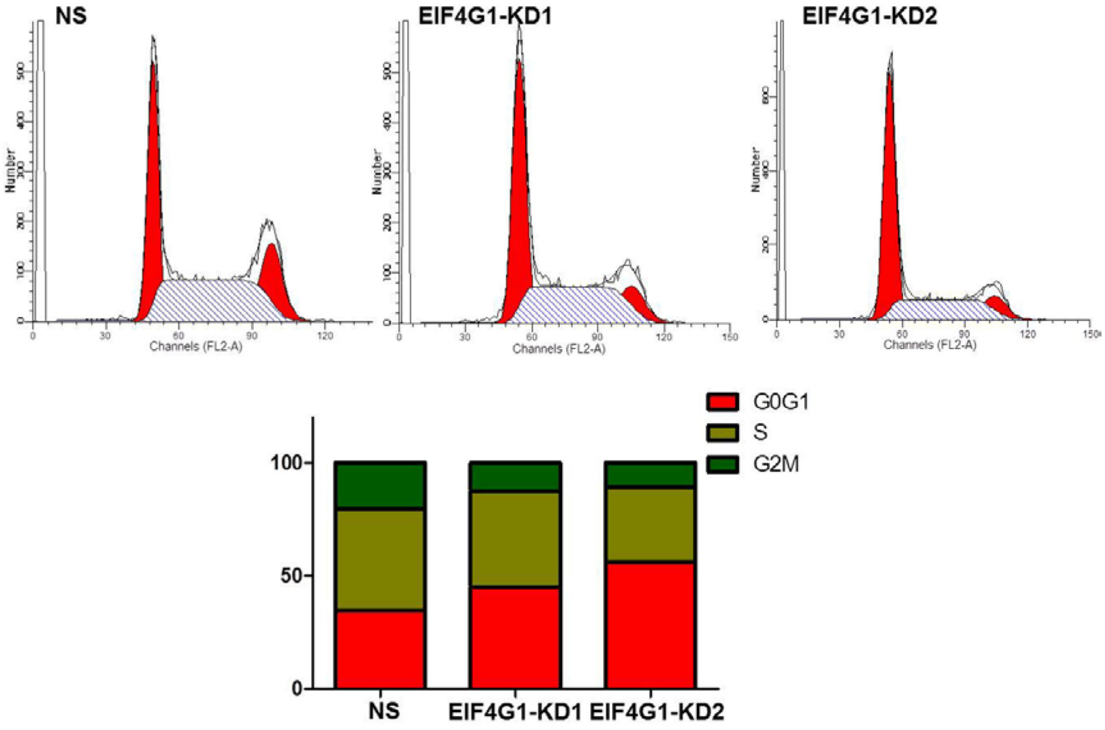

Supplementary Figure S2: Targeting EIF4G1 induces H1299 G0/G1 cell cycle arrest. Cell cycle of stably EIF4G1 "knock-down" H1299 cells (EIF4G1-KD1 and KD2) and control (NS) was determined by PI staining and flow cytometry analysis.

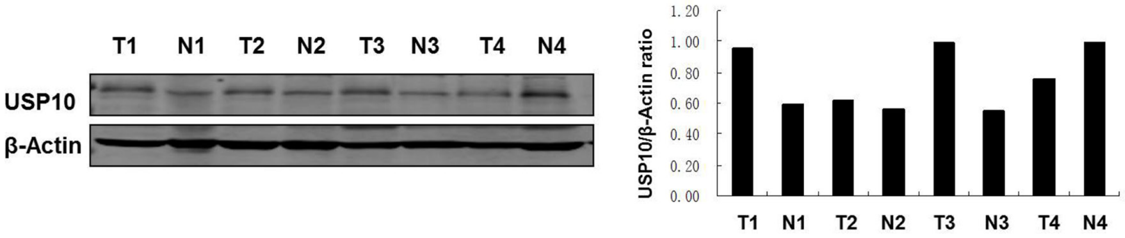

Supplementary Figure S3: The representative immunoblots for USP10 expression in NSCLC tumor tissues and controls. Representative immunoblot results for USP10 expression in tumor tissues (T) and adjacent normal tissues (N) collected from 4 NSCLC patients were shown. The protein band density was quantitated using Image-J software.
